# Drosophila Dyrk2 Plays a Role in the Development of the Visual System

**DOI:** 10.1371/journal.pone.0076775

**Published:** 2013-10-11

**Authors:** Nathan Luebbering, Mark Charlton-Perkins, Justin P. Kumar, Stephanie M. Rollmann, Tiffany Cook, Vaughn Cleghon

**Affiliations:** 1 Department of Developmental Biology, Cincinnati Children’s Hospital Medical Center, Cincinnati, Ohio, United States of America; 2 Department of Pediatric Opthalmology, Cincinnati Children’s Hospital Medical Center, Cincinnati, Ohio, United States of America; 3 Department of Biological Sciences, University of Cincinnati, Cincinnati, Ohio, United States of America; 4 Department of Biology, Indiana University, Bloomington, Indiana, United States of America; University of California, Los Angeles, United States of America

## Abstract

The DYRKs (dual-specificity tyrosine phosphorylation-regulated kinases) are a conserved family of protein kinases that are associated with a number of neurological disorders, but whose biological targets are poorly understood. *Drosophila* encodes three Dyrks: minibrain/Dyrk1A, DmDyrk2, and DmDyrk3. Here we describe the creation and characterization of a *DmDyrk2* null allele, *DmDyrk2^1w17^*. We provide evidence that the smell impaired allele *smi35A^1^*, is likely to encode DmDyrk2. We also demonstrate that *DmDyrk2* is expressed late in the developing third antennal segment, an anatomical structure associated with smell. In addition, we find that *DmDyrk2* is expressed in the morphogenetic furrow of the developing eye, that loss of *DmDyrk2* in the eye produced a subtle but measurable defect, and that ectopic *DmDyrk2* expression in the eye produced a strong rough eye phenotype characterized by increased secondary, tertiary and bristle interommatidial cells. This phenotype was dependent on DmDyrk2 kinase activity and was only manifest when expressed in post-mitotic non-neuronal progenitors. Together, these data indicate that *DmDyrk2* is expressed in developing sensory systems, that it is required for the development of the visual system, and that the eye is a good model to identify *DmDyrk2* targets.

## Introduction

DYRKs are a newly emerging family of protein kinases first recognized as a distinct group in 1996 [1]. DYRKs autophosphorylate an essential tyrosine residue in their activation loop and phosphorylate their substrates on serine and threonine residues [1], [2], [3]. The Dyrks are an evolutionarily ancient family with members present in all eukaryotic supergroups for which genome sequences are available [4]. The Dyrk family, part of the CMGC (cyclin-dependent kinase, mitogen activated protein kinase, glycogen synthase kinase, CDC-like kinase) group of protein kinases, can be subdivided into five categories or subfamilies, Class 1, Class 2, Yaks, HIPKs, and Prp4s [4], [5]. Class 1 and 2 Dyrks represent the original, best-characterized DYRK family members, and will hereafter be referred to as the Dyrks. Drosophila encodes a single Class 1 member, minibrain (Mnb)/Dyrk1A, and two Class 2 family members, smi35A/DmDyrk2 and DmDyrk3 [6].

Class 1 Mnb/Dyrk1A, is a prototype family member first described in *Drosophila* [7]. Both Mnb/Dyrk1A and its mammalian ortholog, Dyrk1A, play critical roles in a variety of processes, particularly neurogenesis in the optic lobes and central brain hemispheres [7], [8], [9]. Loss of both copies of the gene in the fly or one copy in the mouse produces remarkably similar phenotypes – a dramatic reduction in the size of the brain (50% normal size) with accompanying loss of mental faculties, and an overall reduction in the size of the animal (80% normal). Humans are similarly affected, with complete or partial loss of one allele of Mnb/Dyrk1A producing a distinctive clinical syndrome involving primary microcephaly, short stature, intrauterine growth retardation, facial dysmorphism and mental retardation [10], [11], [12]. Mnb/Dyrk1A is also implicated in a number of neurodegenerative diseases including Alzheimer’s, Parkinson’s, and dementia of Lewy bodies [13], [14]. Additionally, Mnb/Dyrk1A is located on human chromosome 21 and is viewed as a significant contributor to the pleiotropic effects of Down Syndrome (DS), also known as trisomy 21, including microcephaly, mental retardation, and early onset Alzheimer’s [15], [16], [17].

In contrast to Mnb/Dyrk1A, very little is known about the *in vivo* functions of DmDyrk2 and Dyrk3. We previously identified DmDyrk2 in a screen for novel tyrosine kinases using an anti-phosphotyrosine antibody-based screen of a *Drosophila* embryonic expression cDNA library. In this initial characterization of the gene, we demonstrated that DmDyrk2 was expressed and was active as a kinase at all stages of the Drosophila life cycle with particular high levels of expression during the embryonic and pupal stages [6].

**Figure 1:**
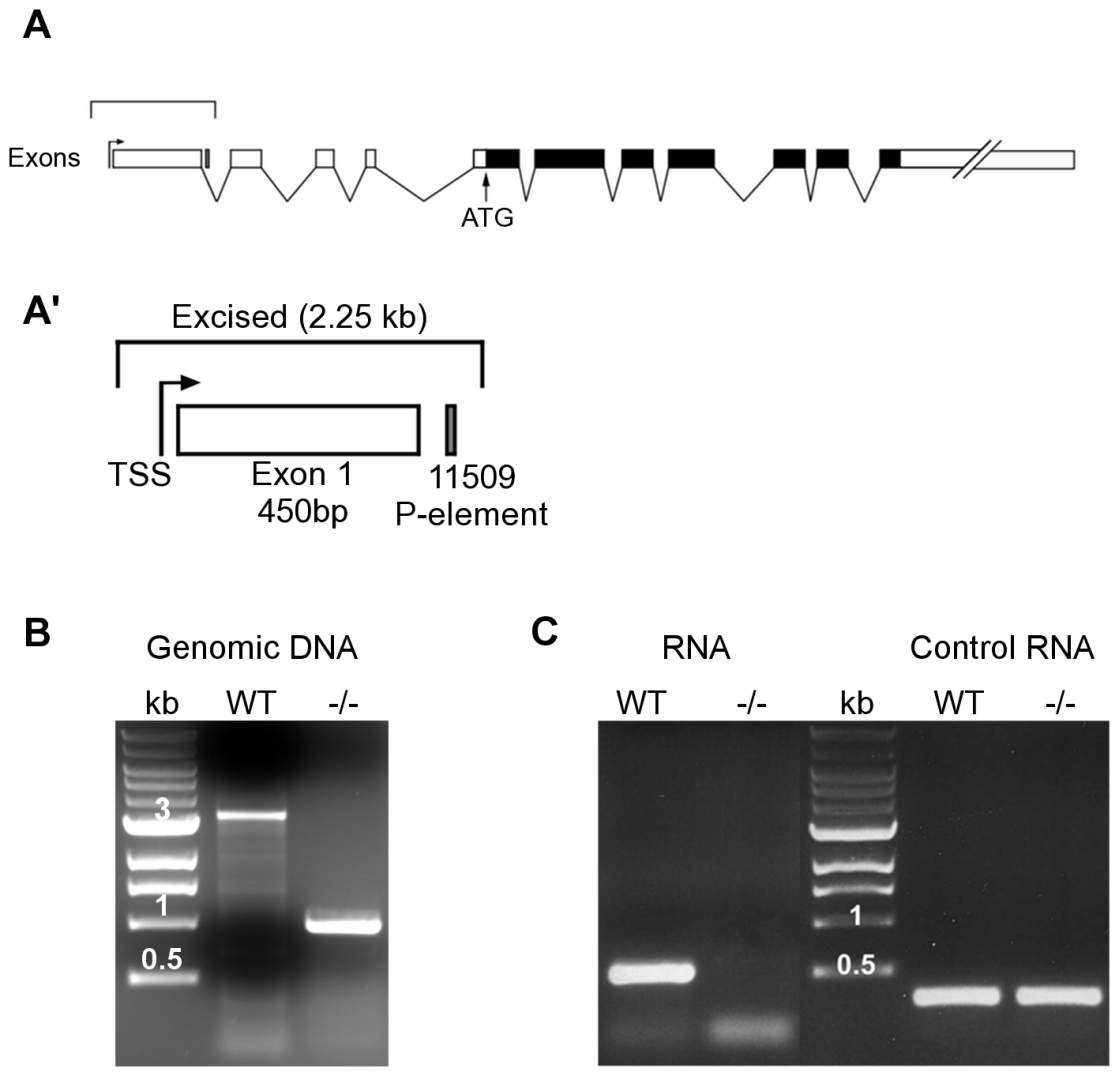
Imprecise excision of P-element generates a *smi35A/DmDyrk2* null allele. (A) Schematic of the *smi35A/DmDyrk2* genomic region (not drawn to scale) showing exons (enclosed boxes), introns (single lines joining exons), and the CDS (black boxes). The relative position of the initiating ATG codon is shown. An upper bracket indicates the region of the gene enlarged in A’. (A’) The insertion site of the *11509* P-element is shown relative to the first exon and predicted TSS of *smi35A/DmDyrk2*. (B) PCR analysis was performed on genomic DNA from WT and *DmDyrk2^−/−^* animals using primers which span the *1w17* deletion. A 1 kb DNA ladder (kb) is shown for comparison with 3, 1 and 0.5 kb sized fragments indicated above the respective band. (C) RT-PCR analysis was performed on RNA isolated from WT and *DmDyrk2^−/−^*0–24 hr embryos using CDS exon-specific primers. An expected 520 bp PCR product is evident in the WT, and is absent in the *DmDyrk2^−/−^* sample. PCR primers specific to the constitutive ribosomal gene rp49 were used as a positive control. A 1 kb DNA ladder (kb) is shown for comparison with 1 and 0.5 kb sized fragments indicated above their respective bands.

DmDyrk2 is the putative protein product of the smell impairment mutant *smi35A* but this issue has not been fully resolved. Initially identified in a P-element based olfactory screen, *smi35A* refers to a P-element insertion in the 35A region of the second chromosome [18]. In a footnote of a separate report, smell impairment was also attributed to two additional P-element insertions (*k16716* and *k06901*) mapping within 21 bp of the original site. These three P-elements are located only ∼20 bp upstream of the wing blister (*wb*) gene and nearly 29 kb from *smi35A/DmDyrk2*. However, the strongest smell-impaired phenotype was associated with *k11509* flies, containing a P-element insertion in the first intron of the *DmDyrk2* transcription unit (Fig. 1A). Thus this gene was designated as *smi35A* [19]. The smell impairment of *smi35A^k11509^*, however, was never formally reported, and the phenotype was subsequently lost [6].

To better understand the *in vivo* role of *DmDyrk2,* we created a null allele of the gene. We show that *DmDyrk2* null flies exhibit a smell-impaired phenotype and that *DmDyrk2* is expressed in the third antennal segment, a structure associated with smell; indicating that *DmDyrk2* encodes the *smi35A* gene. We further demonstrate that *DmDyrk2* is expressed in the morphogenetic furrow (MF) of the developing eye, and that *DmDyrk2* mutants exhibit a reduction in phototransduction activity. In addition, we find that ectopic expression of *DmDyrk2* in post-mitotic eye progenitors produces a rough eye phenotype characterized by extra secondary, tertiary, and bristle interommatidial cells.

## Results

### Generation of a *smi35A/DmDyrk2* Null Allele

Several P-element insertions are associated with *smi35A* [18], [19], but only one, *l(2)k11509*, is located within the *DmDyrk2* transcriptional unit (Fig. 1A and 1A’). To generate a *DmDyrk2* null allele, we re-mobilized *l(2)k11509*, and used PCR analysis to screen for imprecise excision events that deleted the P-element and neighboring genomic sequences. The *DmDyrk2* locus spans nearly 50 kb, with *l(2)k11509* located within its first intron, 540 bp downstream of a single predicted transcriptional start site (TSS) [20], [21] and ∼36 kb upstream of its coding sequence (CDS). Therefore, to generate a transcriptional null allele, we focused on deletions that removed regions upstream of the original P-element insertion site and selected the most extensive, *DmDyrk2^1w17^*, for further characterization. PCR primers spanning the deleted region were identified and used to examine genomic DNA from WT and *DmDyrk2^1w17^* animals. PCR analysis using these primers generated a 3.3 kb product from WT samples and a smaller fragment of ∼1 kb from *DmDyrk2^1w17^* (Fig. 1B). Sequence analysis of the 1 kb product revealed that the *DmDyrk2^1w17^* allele contains a 2,248 bp deletion that removes 1,170 bp upstream and 1,078 bp downstream of the TSS including the entire first exon (Fig. 1A). The only annotated gene in this region is *smi35A*. Like *smi35A^1^* and *smi35A^11509^*, *DmDyrk2^1w17^* is homozygous viable with no obvious visible phenotype.

To verify that *DmDyrk2^1w17^* is a transcriptional null allele, we performed RT-PCR from WT and *DmDyrk2^1w17^* embryos (0–24 hr) and pupae (8 hr after puparium formation [APF]), developmental times of maximum *DmDyrk2* expression [6], using exon-spanning primers. We isolated *DmDyrk2* transcripts in WT but not in *DmDyrk2^1w17^* embryos (Fig. 1C) and pupae (not shown). Furthermore, RNAseq analysis of whole *DmDyrk2* mutant embryos failed to detect any *DmDyrk2* transcripts (data not shown), indicating that no cryptic TSS are utilized. These results suggest that the *DmDyrk2^1w17^* deletion creates a transcriptional null allele, hereafter referred to as *DmDyrk2^−/−^*.

### *Smi35A/DmDyrk2* Null Flies Exhibit a Reduced Smell Avoidance Phenotype

**Figure 2:**
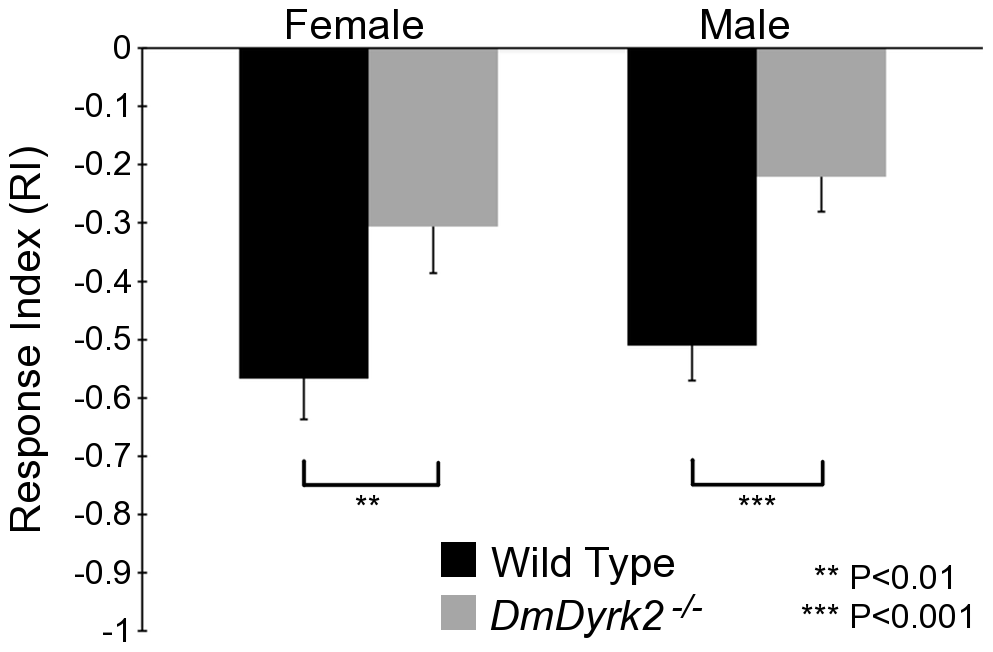
*DmDyrk2^−/−^* animals display a smell impaired phenotype. As described in the text, a T-maze assay was used to determine an RI value for WT and *DmDyrk2^−/−^* male and female adult flies. The results with accompanying standard error bars are shown.

*DmDyrk2* is the putative gene product of the *smi35A* gene [19]; however, the direct attribution of this olfaction deficit to *DmDyrk2* has not been fully addressed (see Introduction). Due to these ambiguities, we made use of *DmDyrk2^−/−^* flies to re-examine this issue. To monitor smell avoidance, we employed a T-maze assay using 1% benzaldehyde as the odorant, the same odorant and concentration used in previous studies [18], [19]. Adult WT and *DmDyrk2^−/−^* flies were given a choice between air without odorant, and air containing the odorant benzaldehyde (Fig. 2). A response index (RI) was then calculated with a positive value indicating preference for the odorant and a negative RI value representing aversion. The RI value for WT flies was ∼−0.5, reflecting a known avoidance of flies for benzaldehyde [18]. Compared to WT, *DmDyrk2^−/−^* animals exhibited a significant “impairment” in benzaldehyde olfactory avoidance behavior (RI of ∼ −0.25 to −0.3). This result, using a T-maze assay, is highly similar to results previously reported for *smi35A^1^* where a simpler behavioral assay was used [18], and suggests that the *DmDyrk2^−/−^* mutation affects the same gene as *smi35A^1^*. Finally, to confirm that flies were not impaired in their ability to move, the locomotor reactivity of WT and *DmDyrk2^−/−^* flies were compared (see Methods). The two populations did not significantly differ in activity (P = 0.56).

**Figure 3:**
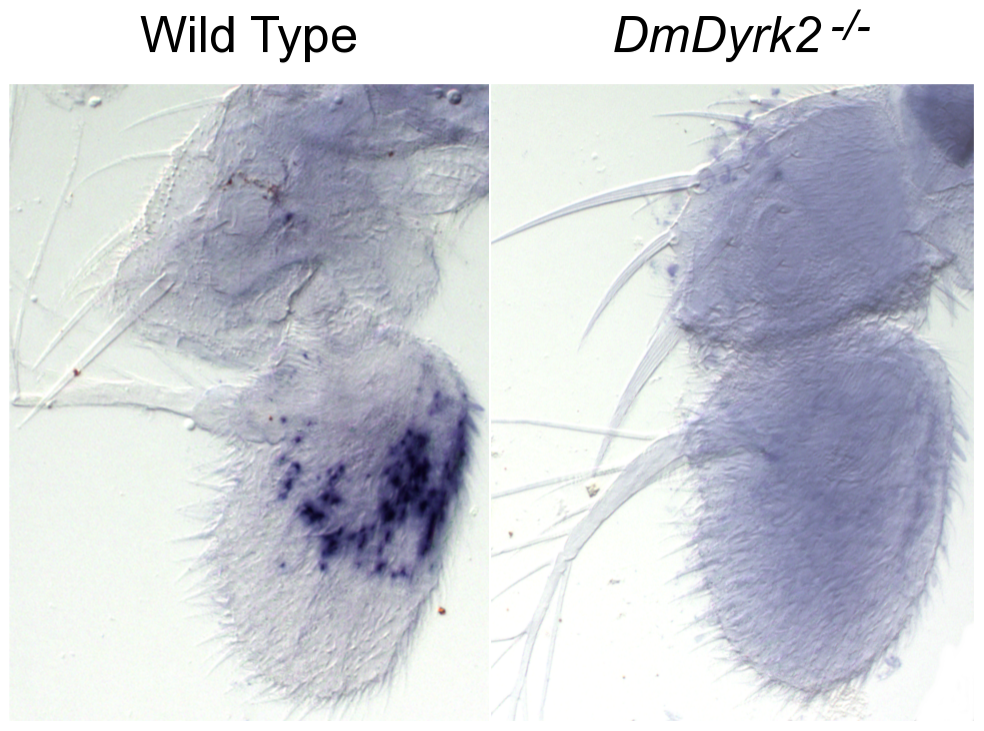
*DmDyrk2* is expressed in the third antennae segment. Antennae were dissected from late pupae (65 hr APF) of WT and *DmDyrk2^−/−^* flies. Specific probes were then used to examine the structures for the presence of *DmDyrk2* transcripts. Evidence of *DmDyrk2* expression is only seen in the third antennal segment of WT flies.

The Drosophila olfactory response is mediated by odorant receptors expressed in olfactory sensory neurons located within sensilla on the third antennal segment and maxillary palps [22]. Previous enhancer trap analysis of *smi35A^1^* reported but did not demonstrate β-galactosidase expression in the third antennal segment [18]. To directly determine if *smi35A/DmDyrk2* transcripts were present in the third antennal segment, we performed *in situ* hybridization. We show here that *DmDyrk2* expression was observed in the third antennal segment of late pupae from WT but not *DmDyrk2^−/−^* animals (Fig. 3). We saw no reproducible difference in staining in other parts of the antenna. From these experiments, we conclude that *DmDyrk2* is expressed in the third antennal segment and that loss of its expression results in impaired odor-guided smell avoidance.

### *DmDyrk2* is Expressed in the Morphogenetic Furrow of the Eye Imaginal Disc

**Figure 4:**
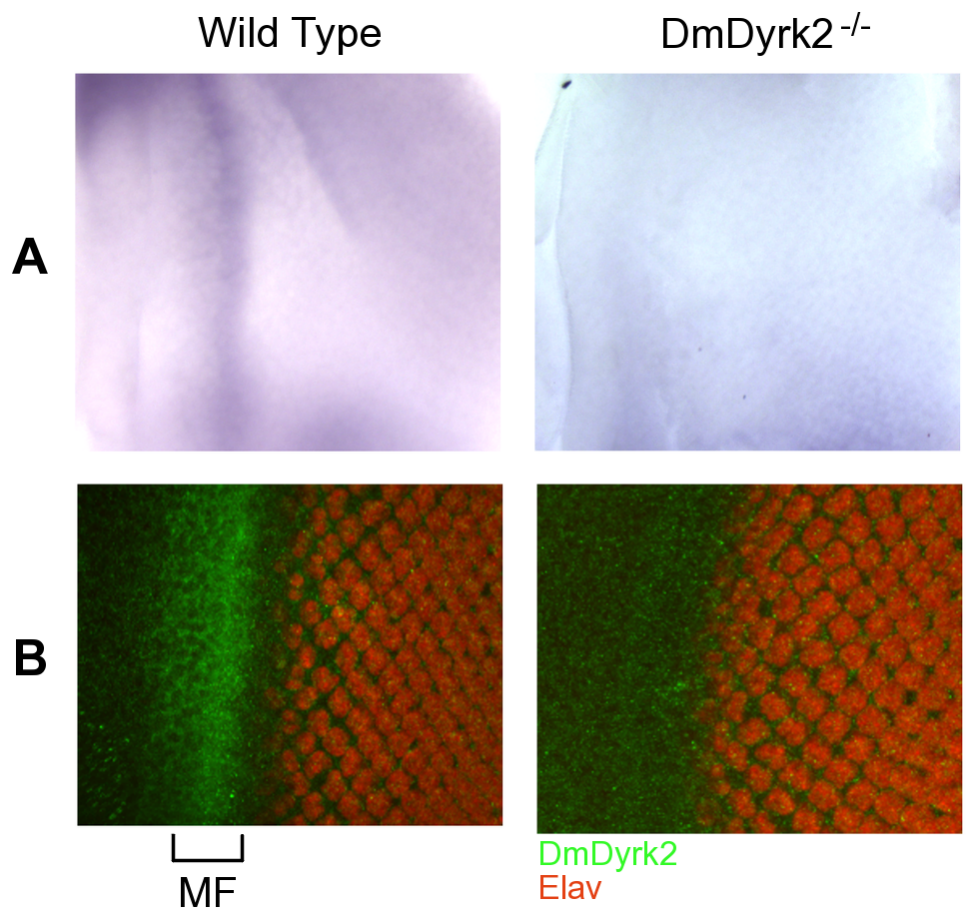
*DmDyrk2* is expressed in the MF of developing eye imaginal discs. (A) Eye discs from WT or *DmDyrk2^−/−^*3^rd^ instar larvae were probed for the presence of *DmDyrk2* mRNA by in situ hybridization. (B) Specific antibodies were used to probe similar discs for the presence of *DmDyrk2* (green) or Elav (red). The morphogenetic furrow (MF) is indicated by a bracket. Expression of *DmDyrk2* mRNA and protein can be seen in this region of WT but not *DmDyrk2^−/−^* samples. DmDyrk2 expression can be seen in the MF slightly ahead of Elav-expressing, terminally differentiated neuronal cells.

The eye-antennae imaginal disc is a cluster of undifferentiated cells set aside during embryogenesis that are destined to form the adult eye and antennae. During the first two and one-half stages of larval development, cells in the eye-antennae imaginal disc proliferate, with differentiation initiating in the late stages of the last (third) larval stage [23]. During analysis of DmDyrk2 expression, we examined *DmDyrk2* RNA and protein expression in eye-antennae discs from WT and *DmDyrk2^−/−^* late 3^rd^ instar larvae. We did not unambiguiously detect *DmDyrk2* expression in the antennae portion of the discs at these early stages. However, *DmDyrk2* mRNA expression is clearly evident at the boundary between proliferation and differentiation – the morphogenetic furrow in developing wild-type eye discs, expression that is absent in *DmDyrk2^−/−^* discs (Fig. 4). We also analyzed protein expression using a DmDyrk2-specific antibody [6] and confirmed that DmDyrk2 protein was observed in the morphogenetic furrow of WT but not *DmDyrk2^−/−^* eye-antennal discs (Fig. 4).

**Figure 5:**
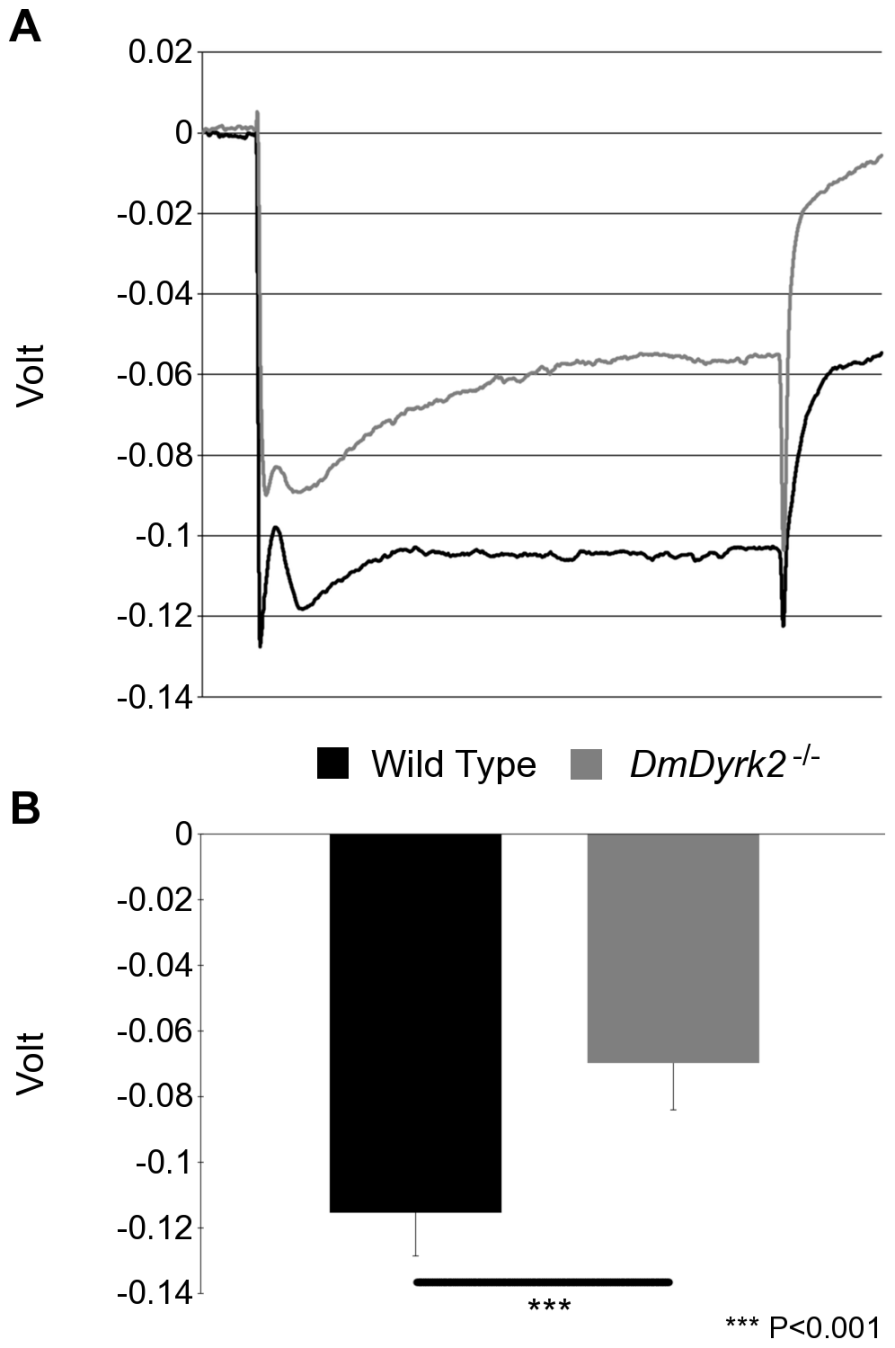
WT and *DmDyrk2^−/−^* flies exhibit distinct ERG profiles. (A) Example of a single ERG recording from a 5 sec light pulse from WT and *DmDyrk2^−/−^* flies. (B) Maximal photoreceptor response of WT and *DmDyrk2^−/−^* flies.

Similar to a lack of external antennal defects in *DmDyrk2^−/−^* flies, we did not detect an obvious eye phenotype in *DmDyrk2^−/−^* flies by light or scanning electron microscopy. To assess whether *DmDyrk2^−/−^* flies exhibited impairment in visual integrity, we performed electroretinograms (ERGs). In wild-type (WT) flies, photoreceptors exhibit a strong depolarization in response to light stimulation, calculated as the maximum photoreceptor response [24]. As shown in Fig. 5, there was a significant reduction in photoreceptor depolarization in the ERG profile of WT and *DmDyrk2^−/−^* compared to WT flies (Fig. 5).

### Ectopic *DmDyrk2* Expression in Post-mitotic Retinal Progenitors Causes Patterning Defects

**Figure 6:**
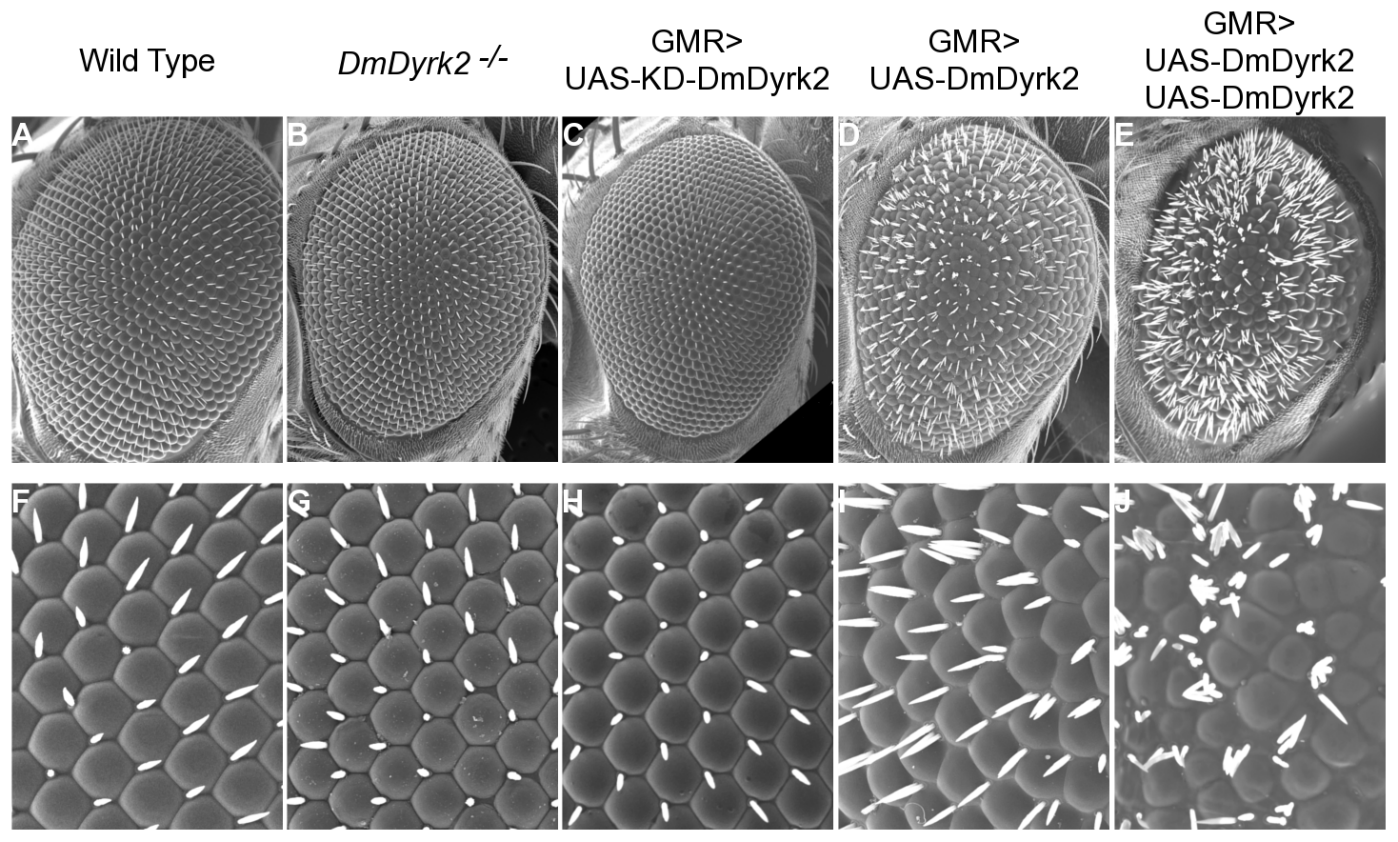
Overexpression of DmDyrk2 in cells following the MF produces a rough eye phenotype. SEM was used to visualize adult eyes from the following genotypes: WT (A and F), *DmDyrk2^−/−^* (B and G), *GMR-GAL4/+; UAS-DmDyrk2^KD^/+* (C and H), *GMR-GAL4/UAS-DmDyrk2^WT^* (D and I), and *GMR-GAL4, UAS-DmDyrk2^WT^/UAS-DmDyrk2^WT^* (E and J).

To gain further insight into *DmDyrk2* function, we ectopically expressed *DmDyrk2* in eye progenitors using the GAL4-UAS system [25]. FLAG-tagged WT and kinase inactive (KD) DmDyrk2 cDNAs (*Dyrk2^WT^* and *Dyrk2^KD^*) were cloned downstream of GAL4-responsive UAS sites, and transgenic flies were generated by P-element-mediated transformation. We then expressed UAS-DmDyrk2 using several eye-specific GAL4 driver lines and examined adult eye morphology using scanning electron microscopy. Ectopic expression of Dyrk2^WT^ with *eyeless-GAL4* showed no effect on patterning (data not shown), while misexpressing Dyrk2^WT^ following the MF using *GMR-GAL4* (GMR>Dyrk2^WT^) produced rough eyes with extra and/or misplaced interommatidial bristles (Fig. 6). This phenotype was observed using 6/6 independent Dyrk2^WT^ insertion lines. Flies carrying two copies of the DmDyrk2^WT^ showed an even more severe phenotype, with eyes being smaller than controls, individual ommatidia difficult to discern, and a pronounced increase in bristle formation (Fig. 6).

A similar phenotype, although to a lesser extent, was observed when Dyrk2^WT^ was expressed using *sevenless-GAL4* to drive expression in a subset of cells following the MF (not shown). Both the *GMR-* and *sevenless-GAL4* associated phenotypes were dependent on DmDyrk2 kinase activity, as no phenotype was observed when kinase inactive Dyrk2^KD^ was expressed using the same GAL4 driver lines (3/3 independent KD-DmDyrk2 insertion lines tested) (Fig. 6), indicating that DmDyrk2 requires kinase activity for this gain of function phenotype. Finally, no phenotype was observed when Dyrk2^WT^ was expressed in more differentiated neuronal (*Elav-GAL4*), photoreceptor (*otd-GAL4*), or cone (*spa-GAL4*) cells (not shown).

**Figure 7:**
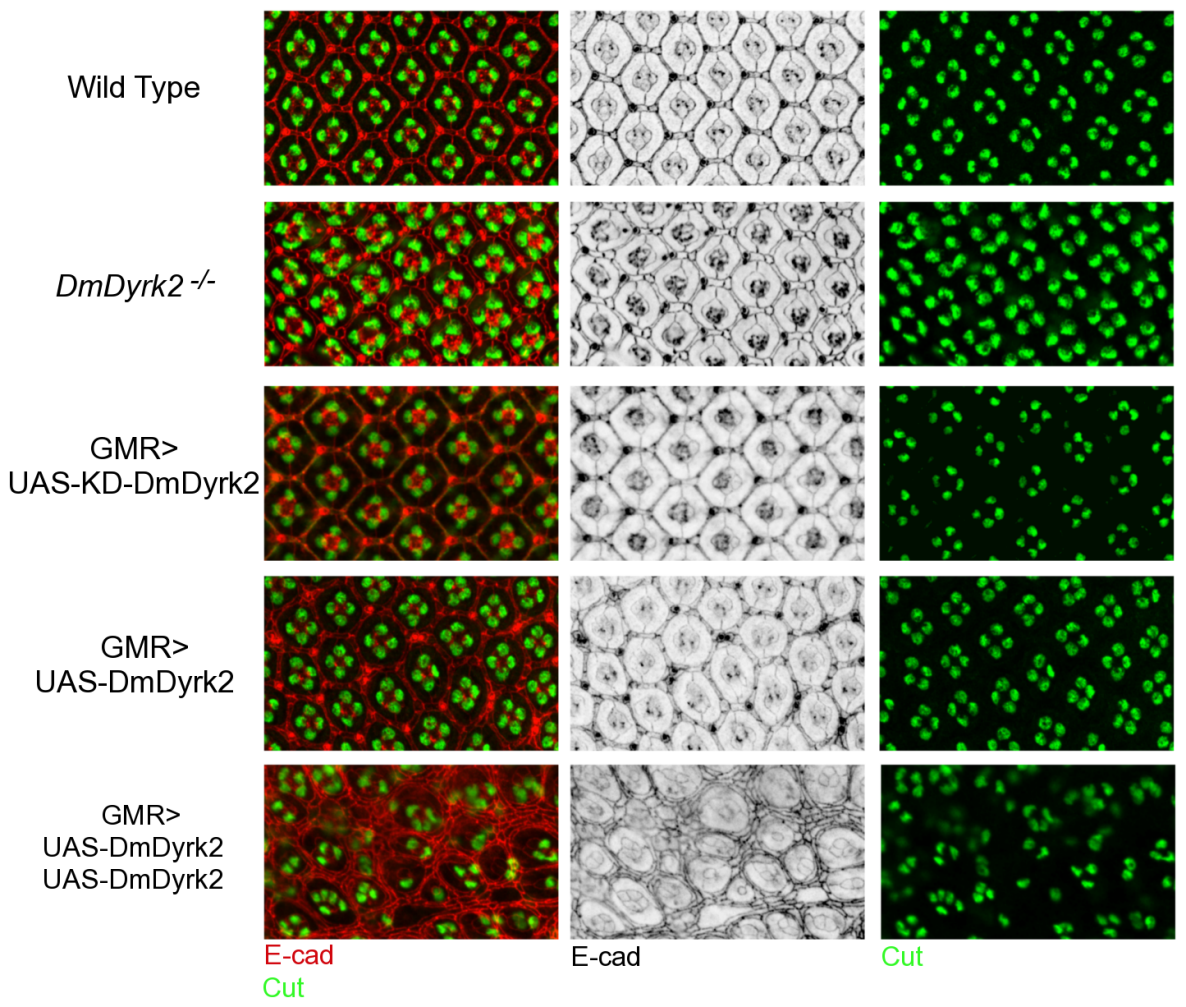
Immunofluorescent confocal analysis of mid-pupal DmDyrk2 loss- and gain-of-function retinas. Pupal retinas were dissected 45-cadherin (red, left panels; black, middle panels) to mark apical cell membranes, and Cut (green, left and right panels) to mark cone cell nuclei. Overlays are represented in left panels. Genotypes are: wild type (Canton S), *DmDyrk2^−/−^* (*1w17/1w17)*, GMR>UAS-KD-DmDyrk2 (*GMR-GAL4/+*, *UAS-DmDyrk2^KD^/+*), GMR>UAS-DmDyrk2 (*GMR-GAL4/UAS-DmDyrk2^WT^*), and GMR>UAS-DmDyrk2-UAS-DmDyrk2 (*GMR-GAL4, UAS-DmDyrk2^WT^/UAS-DmDyrk2^WT^*).

Eye patterning is complete by ∼40% pupation. Therefore, to further characterize the GMR>Dyrk2^WT^ phenotype, we analyzed pupal retinas for the presence and pattern of known cell types. Dissected retinal tissue was immunostained with antibodies against Cut, a marker of the four lens-secreting cone cells, and E-cadherin which recognizes apical cell surfaces. E-cadherin staining of WT discs shows the intricate latticed symmetry of the compound eye with an organized cone cell quartet surrounded by two primary pigment cells in the center of each ommatidium, bordered by a single layer of secondary, tertiary and bristle cells shared with neighboring ommatidia (Fig. 7, top row). Consistent with our SEM results, pupal retinas from *DmDyrk2^−/−^* and GMR>Dyrk2^KD^ flies did not display an overt phenotype (Fig. 7, second and third row, respectively). However, we never observe as regular a lattice array as seen in WT eyes, suggesting the possibility of a subtle phenotype. GMR>Dyrk2^WT^ flies, on the other hand, showed an obvious disruption in patterning (Fig. 7, fourth row) with an increase and/or misplacement of secondary, tertiary, and inter-ommatidia bristle cells. In addition, although cone cell number remains unchanged, slight ommatidial rotation defects were observed. Eye discs from animals carrying two copies of the Dyrk2^WT^ transgene showed an even more severe disruption in patterning with numerous secondary, tertiary and bristle cells separating each ommatidium (Fig. 7, bottom row). Cone cell patterns were also disturbed and the overlay of E-cadherin and Cut staining revealed that cone cell number was also affected with 1, 2, 3 and even 5 cone cells present in an individual ommatidium (Fig. 7, bottom row).

Combined, these results indicate that *DmDyrk2* mRNA and protein are expressed at the morphogenetic furrow, and that overexpressing *DmDyrk2* in cells after the morphogenetic furrow severely alters eye development.

## Discussion

Here we describe a null *DmDyrk2* allele, generated by imprecise P-element excision. *DmDyrk2^−/−^* mutants are homozygous viable with no overt visible phenotype, but do display impairments in both olfactory behavior and vision. In addition, ectopic DmDyrk2 expression in the eye leads to severe mispatterning defects. Together, these data indicate that like Mnb/Dyrk1A, DmDyrk2 is critical for nervous system development. In olfactory tests, *DmDyrk2^−/−^* flies exhibit a smell impaired phenotype with reduced response to the aversive odorant, benzaldehyde (Fig. 2). Consistent with a role in smell, we found that *DmDyrk2* was expressed in the third antennal segment of the fly (Fig. 3), a structure associated with odorant detection [22]. These results indicate that the *DmDyrk2* transcriptional unit is responsible for the *smi35A* phenotype described earlier [18]. A variety of factors may account for the observed smell impaired phenotype and future work will be needed to determine the exact cause.

In this study, we also report that *DmDyrk2* RNA and protein are expessed in the MF of developing eye discs (Fig. 4). While we did not observe an obvious eye phenotype in *DmDyrk2^−/−^* flies by SEM or by sectioning (see below), ERG experiments showed a significant deficit in photoreceptor responses in *DmDyrk2^−/−^* flies (Fig. 5). Given that the MF precedes photoreceptor differentiation by days, it is possible that the lack of a developmental phenotype in *DmDyrk2* mutants is due to redundancy with other Dyrk family members. For example, based on genome-wide expression analysis *DmDyrk3* is highly expressed in the developing eye-antennal disc and in adult flies [26]. Functional redundancy between Dyrk family members Hipk1 and Hipk2 has previously been documented; mice lacking either Hipk1 or Hipk2 are viable and fertile; however, loss of both genes is embryonic lethal [27]. Therefore, future experiments aimed at understanding DmDyrk3 function, and defining potential overlapping functions with DmDyrk2 should be informative regarding how the Dyrks function during neurogenesis.

Although the cellular and molecular basis for the reductions in vision and olfactory responses due to loss of *DmDyrk2* remains unclear, there is precedent for a Dyrk family role in these sensory processes. Overexpression of Dyrk family member Mbk1 in the nematode *Caenorhabditis elegans* leads to defects in olfaction [28], perturbation of Mnb/Dyrk1A function in the fly leads to defects in both smell and vision [7], and loss of function of Mnb/Dyrk1A in mice results in defects in retinal development [9].

To gain further insight into the role of *DmDyrk2* in eye development we have used the GAL4-UAS binary system to ectopically express the gene in the developing eye. Terminal differentiation of ommatidia in the compound eye occurs as a wave. The leading edge of the wave is visible as an indentation termed the MF which moves posterior to anterior across the eye disc. In front of the MF, unpatterned cells divide freely. With passage of the MF, cells arrest in the G1 phase of the cell cycle and patterning begins within the furrow. As the furrow progresses, cells within and behind the MF are recruited into photoreceptor clusters in an ordered sequence. After passage of the MF, cells which have not differentiated as photoreceptors reenter S phase and divide a final time, the so called “second mitotic wave”. Pattern formation then continues as more cells are added to form fully differentiated eyes [29], [30].

Eye development therefore exhibits several discrete temporal events separated in time and space by passage of the MF. We used several eye-specific GAL4 driver lines to express DmDyrk2 in discreet temporal and developmental subpopulations: *Eyeless-GAL4*, drives expression of UAS-genes in all cells in front of the MF (non-differentiated, non-synchronous, proliferating cells); *GMR-GAL4* drives expression in all cells following the MF (cells undergoing differentiation with limited proliferation), *sevenless-GAL4* drives expression in a subset of cells following the MF (photoreceptor precursors and certain nonneuronal cells such as cone cells with limited proliferation); and *elav-* or *Otd-GAL4* drive expression in non-dividing, fully differentiated cells behind the MF (neuronal cell lineage and photoreceptor cells, respectively) [31], [32], [33].

This analysis revealed that expression of the WT gene had profound effects on eye development but only when expressed with GAL4 driver lines that were active in cells within or immediately behind the MF (ie, *GMR-* and *sevenless-GAL4*). The absolute dependence on the intact kinase activity of DmDyrk2 for the observed phenotypes when the protein is expressed under the control of *GMR-* or *sevenless-*GAL4 indicates the effects are mediated through the phosphorylation of specific substrates. Our results indicate that DmDyrk2 mRNA and protein are expressed at a location (MF) and time (late larva) in development when the eye is patterned; and that overexpression of the gene in this time and place severely alters normal eye development. Furthermore, the eye phenotype requires developmental changes initiated by passage of the MF. These results are consistent with the premise that signaling pathways and/or substrates impacted by Dyrk expression are not present, or are not functional, except during a critical period after cells have committed to a particular cell fate with limited proliferative ability but prior to terminal differentiation. Notably, several Dyrk family members function in other cells undergoing a transition from growth to differentiation including the Dictyostelium Dyrk-family member YakA, and vertebrate MIRK/Dyrk1B and Drosophila Mnb/Dyrk1A [34], [35], [36], [37]. In addition, Dyrk family members impact a variety of processes consistent with our observations including cell survival, apoptosis, differentiation, and cell cycle control [5], [38].

## Materials and Methods

### Fly Lines

*Drosophila melanogaster* lines were maintained used standard husbandry procedures. Canton S flies were used as wild-type, *GMR-GAL4* line 816 has been described previously [39], *spa-GAL4* (Bloomington stock 26656), *eyeless-Gal4* (Bloomington stock 5535), *sevenless-Gal4* (Bloomington stock 2023), *otd-Gal4* [33], and *elav-Gal4* (Bloomington stock 458).

### Generation of a *DmDyrk2* Null Allele

The *smi35A/DmDyrk2* null allele was generated by using the genomic copy of delta2-3 transposase at 99B to mobilize the *P{lacW}* element in *k11509* flies, and screening for imprecise excision events. Complete or partial loss of the P-element was first screened through loss of the *P{lacW}*-associated eye color, and then by PCR to identify imprecise excision events which also deleted genomic regions of *smi35A*. Twenty-seven such lines were isolated, two of which contained deletions removing genomic regions 5′ to the original P-element insertion. The larger of these two 5′-deficiencies, 1w17, was analyzed further. PCR analysis using the primer pair 5′-GCAGAATCTTTGAGTAGACAATCCG-3′ [3965For] and 5′-GGTCTCTGACAACTTCAAGTTGATGGTGG-3′ [55021Rev] spanned the deletion. These primers were used to amplify the region containing the deletion and the PCR product was sequenced to determine the exact boundaries of the deletion.

### RT- PCR

Total RNA was extracted from WT and *Dyrk2^1w17^* 0–24 hr embryos using a QiaShredder and an RNeasy mini kit (Qiagen). Primers specific to the DmDyrk2 CDS regions, exon 9 and 11 (5′-TTGAACCTGTACGAATTGAT-3′ [Exon9For] and 5′-ACTTGCTGCCTGGCGACCGT-3′ [Exon11Rev]), were used to analyze samples by PCR. This region is unaffected in the 1w17 mutant. Products derived from wild-type DmDyrk2 transcripts or from genomic DNA generate 520 or 2,289 bp fragments, respectively. Primers specific to the constitutive ribosomal gene rp49 (5′-TACAGGCCCAAGATCGTGAA-3′ [rp49For] and 5′-TCTCCTTGCGCTTCTTGGA-3′ [rp49Rev]) were used as an endogenous control.

### Antennal Dissection and In-Situ Hybridization

Antennal segments attached to the head casing were dissected from pupae (65 hr APF) grown at 25°C. Samples were fixed and hybridized with a probe made against DmDyrk2 following the ‘In Situ Hybridization’ protocol (Sullivan, W., Ashburner, M., *Drosophila* Protocols, 2000 216–220). DIG labeled RNA probes were made using the DIG RNA Labeling Kit (SP6/T7) (Roche, Cat# 11175025910). The probe was made against FLAG-tagged full length DmDyrk2 in pcDNA3 using SP6 polymerase and was diluted 1∶1000 and developed for 15 min.

### Smell Avoidance Assay

To normalize the genetic backgrounds for olfactory behavioral studies, *DmDyrk2^−/−^* flies were first “Cantonized” by seven rounds of backcrossing to WT Canton-S flies as described in Goodwin et al, [40]. Briefly, *DmDyrk2^−/−^* and Canton-S flies were mated. To allow free recombination, resultant virgin heterozygous female offspring (*DmDyrk2^−/+^*) were then mated to Canton-S males. Individual virgin female offspring were again mated to Canton-S males and PCR was used to screen their progeny for the presence of the 1w17 deletion. After identification of a deletion-positive line, this procedure was repeated five times. After the final backcross, heterozygous *DmDyrk2^−/+^* offspring were allowed to mate, and from their progeny single virgin female-male pairs were established. These lines were then screened for homozygous *DmDyrk2^−/−^* flies using the primer pair 3965For and 55021Rev described above to insure the presence of the 1w17 allele; and the primer pair (5′-GGATTATAAGACGTGGGCTACC-3′ [4551For] and 5′-GTGTGCGTGAGAACTTCTGC-3′ [4869Rev]), present within the deleted region, to confirm absence of the WT allele.

The smell avoidance assay was performed as previously described [41], [42]. Adult WT or *DmDyrk2^−/−^* flies were collected 7 days after eclosion, separated by sex into groups of 30, transferred to new vials, and allowed to recover for a minimum of 24 hr. One hour prior to testing, flies were transferred to empty vials. Flies were placed in a T-maze apparatus, allowed to adjust for 1 min, and then given a choice between airflow (500 mL/min) in the two arms of the T; one arm containing odorant dissolved in paraffin oil, and the second arm containing only paraffin. Based on choice, a response index (RI) was calculated using the formula: RI = (No. flies choosing odor – No. flies choosing control)/(No. total flies); with a positive value indicating preference for the odorant and a negative value aversion to the odorant. The experiment was conducted in two separate runs on different days. For each run, 300 animals of each sex and genotype were tested per experiment in groups of 30 individuals each.

Locomotor reactivity assays were performed on individual WT Canton-S and *DmDyrk2^−/−^* male and female flies for 30 sec according to the methodology described by Jordan et al. [43]. Briefly, mated 3 day old flies were collected and individual flies of either sex were placed into vials containing food for 24 hr. Vials were then tapped 3 times against a hard surface and the time the fly was active over a 30 sec time period was quantitated with a timer. Mean time of activity for WT flies was 25.2 sec (25.1 sec for males, 25.4 sec for females), and 24.8 sec for *DmDyrk2^−/−^* mutants (24.5 sec for males, 25.1 sec for females). Activity values of WT and *DmDyrk2^−/−^* flies were then compared using single factor ANOVA. The two populations did not differ significantly in this behavior (P = 0.56). WT flies were active 84% and *DmDyrk2^−/−^*83% of the time period monitored.

### Electroretinograms

One day old WT and Cantonized *DmDyrk2^−/−^* flies were immobilized with CO_2_, attached to plastic cover slips pink dental wax (Electron Microscopy Sciences) and dark adapted for 30 min. A recording electrode (a cotton wick containing 0.9% NaCl connected to a silver wire) was positioned on the surface of the eye, and the grounding electrode (a glass electrode containing 0.9% NaCl connected to a silver wire) in the abdomen. 5 pulses (5 secs on, 15 sec off) of white light were delivered through an optical fiber connected to an LED. Data were analyzed in MATLAB (MathWorks, Inc., Natick, MA, USA) to calculate averages of the maximal photoreceptor response from each sample. Values represent data from 5 individual flies of each type, with 5 light pulses each; n = 25. Results were compared using single factor ANOVA. The two populations differed significantly (P = 0) in this behavior.

### Creation of UAS-DmDyrk2 Transgenic Flies

UAS-FLAG-DmDyrk2 transgenic flies were created by cloning an EcoR1-EcoR1 fragment containing FLAG-tagged DmDyrk2^WT^ and DmDyrk2^KD^ cDNA [6] into the pUAST vector. Transgenic flies were made by the European Molecular Biology Laboratory (EMBL) by microinjection of *P[w+, UAS_GAL4_hsp70-FLAG-DmDyrk2]* constructs into *w^1118^* embryos with a helper plasmid encoding the delta2-3 P-element transposase to allow integration into the genome. Individual injected animals were screened for the presence of UAS-DmDyrk2 by eye color and subsequently stabilized using the appropriate chromosomal balancers.

### Scanning Electron Microscope

Adult eyes were visualized using flies mounted on carbon tabs and directly analyzed using a Hitachi S-3400N as described previously [44].

### Immunohistochemistry

Imaginal eye discs and pupal retinas from 25°C pupa 45 hours after puparium formation were dissected in 1× PBS and fixed in 1×PBS plus 4% formaldehyde. Discs were stained with antibodies using procedures previously described [44]. Antibodies used for staining were first diluted in 1× PBS as follows: Dyrk2 (rabbit [6], 1∶4,000), Elav (rat, Developmental Studies Hybridoma Bank, 1∶200), Cut (mouse, Developmental Studies Hybridoma Bank, 1∶100), E-cadherin (rabbit, Santa Cruz Biotechnology, 1∶150). Secondary antibodies conjugated to Alexa Fluor or Dylight 488, 555, and 647 nm (donkey, Invitrogen or donkey, Jackson Immunoresearch) were diluted 1∶500. Stained discs were mounted in ProLong Gold containing DAPI (Invitrogen), and samples were imaged using a Zeiss LSM 700 confocal microscope and processed with ImageJ (1.45s, National Institutes of Health, USA, Imagej.nih.gov/ij) and Gimp (v2.6.11, GNU Image Manipulation Program, GIMP.org).
