# Correction: Drosophila Dyrk2 Plays a Role in the Development of the Visual System

**DOI:** 10.1371/journal.pone.0092344

**Published:** 2014-03-14

**Authors:** The PLOS ONE Staff

Pamela A. Lochead is incorrectly omitted from the list of authors. Dr. Lochead should be listed as the fourth author and affiliated with Signalling Programme, The Babraham Institute, Babraham Research Campus, Cambridge, CB22 3AT, England, UK. Dr. Lochead should be indicated in the Author Contributions statement with the initials PL and as having conceived and designed the experiments, performed the experiments and analyzed the data.

The correct Citation is: Luebbering N, Charlton-Perkins M, Kumar JP, Lochead PA, Rollmann SM, et al. (2013) Drosophila Dyrk2 Plays a Role in the Development of the Visual System. PLoS ONE 8(10): e76775. doi:10.1371/journal.pone.0076775
